# Cathelicidin antimicrobial peptide (*CAMP*) gene promoter methylation induces chondrocyte apoptosis

**DOI:** 10.1186/s40246-021-00321-8

**Published:** 2021-04-23

**Authors:** Guoliang Wang, Yanlin Li, Guang Yang, Tengyun Yang, Lu He, Yang Wang

**Affiliations:** Department of Sports Medicine, First Affiliated Hospital of Kunming Medical University, Kunming, 650031 Yunnan China

**Keywords:** CAMP, Methylation, Osteoarthritis, ROS, Inflammatory factors

## Abstract

**Objective:**

The occurrence of osteoarthritis is related to genetic and environmental factors. Among them, the change of chondrocyte gene expression pattern regulated by epigenetic modification is an important participant. This study analyzed the effect of *CAMP* gene methylation on the level of oxidative stress and inflammation of chondrocytes.

**Methods:**

We analyzed the changes of the transcriptome in the articular cartilage tissue of osteoarthritis (OA) patients from the GSE117999 dataset. The GSE48422 dataset was used to analyze the changes in the methylation level of osteoarthritis cells. Cell Counting Kit-8 (CCK-8) and flow cytometry analysis of short hairpin RNA (shRNA) silencing *CAMP* gene and 5-μM 5-Aza-2’-Deoxycytidine (AZA) treatment on the proliferation and apoptosis of Human chondrocytes osteoarthritis (HC-OA) cells. The Dichloro-dihydro-fluorescein diacetate (DCFH-DA) assay was used to detect the level of reactive oxygen species (ROS), and the expression level of inflammatory factors was analyzed by Western Blot.

**Results:**

The expression of CAMP in cartilage tissue of OA patients was upregulated, and the level of methylation was downregulated. CAMP was highly expressed in osteoarthritis articular cartilage cells. Silencing CAMP inhibited the proliferation of HC-OA cells and promoted their apoptosis. *CAMP* gene methylation inhibited ROS levels and tumor necrosis factor-α (TNF-α) expression levels in HC-OA cells, and promoted transforming growth factor beta (TGF-β) expression. *CAMP* gene methylation inhibited the proliferation of HC-OA cells and promoted their apoptosis.

**Conclusion:**

*CAMP* gene promoter methylation inhibits ROS levels and inflammation and induces chondrocyte apoptosis.

## Introduction

Osteoarthritis (OA) is a common age-related degenerative disease. Its main pathological feature is articular cartilage degeneration, which is mainly manifested by the loss of articular cartilage and the formation of osteophytes, subchondral bone sclerosis, and synovium inflammation, among others; clinical symptoms such as pain, joint deformity, and dysfunction may appear in the later clinical stage [1–3].

Research in recent years has shown that epigenetic modification plays an important role in the pathogenesis of OA [4, 5]. Epigenetic modification mainly includes DNA methylation, histone modification, chromatin remodeling, and non-coding RNA, among which DNA methylation is closely related to inflammatory diseases [6, 7]. At present, DNA methylation studies of OA mainly focus on articular cartilage, because articular cartilage is the core tissue involved in the disease process [8]. In addition, DNA methylation has become an important regulator of chondrocyte dedifferentiation, severely destroying the results of autologous chondrocyte implantation in patients with cartilage injury [8]. Therefore, it is of great significance to study the DNA methylation profile of chondrocyte dedifferentiation.

At present, the methylation sites or regions identified in OA genome-wide methylation studies are mostly enriched in genes related to immunity or inflammation, cartilage development, transcription factor regulation, and protease activity [9, 10]. Although OA was once considered a non-inflammatory disease, it has been confirmed that inflammation is very important in the occurrence of OA [11].

Tumor necrosis factor-α (TNF-α) is one of the main pro-inflammatory cytokines related to the pathogenesis of OA; it can stimulate the release of matrix metalloenase-1 (MMP-1), MMP-3, and MMP-13 in OA patients, and inhibit the synthesis of proteoglycans and type II collagen [12]. Transforming growth factor beta (TGF-β) is a member of the TGF-β superfamily. It transmits signals to the nucleus through the TGF-β receptor and its intracellular signaling system to play a variety of different physiological regulatory roles [13, 14]. Studies have shown that TGF-β plays an important role in maintaining normal articular cartilage and joint repair [15].

Some researchers have used the demethylating agent AZA for in vitro intervention of OA chondrocytes. For example, Iliopoulos et al. [16] found that AZA could reduce the methylation level of the *leptin* gene promoter of chondrocytes and increased the expression of *leptin* mRNA, which then activated MMP-13. Kim et al. [17] reported that when OA articular chondrocytes were co-cultured with 10 μM AZA for 8 days, the methylation level of 6 CpG islands in the Sry-type high-mobility-group box 9 *(SOX-9)* promoter region of chondrocytes decreased, and the expression level of SOX-9 increased.

The *CAMP* gene (also known as LL-37) is located on chromosome 3p21.31. The CAMP protein is composed of 37 amino acids in a spiral shape and has a broad-spectrum antibacterial activity. Studies have shown that CAMP has defensive effects, such as regulating inflammation, inducing immune cells to injured or infected sites, binding and neutralizing lipopolysaccharide (LPS), promoting epithelialization, and repairing the damage [18]. Some researchers had found that CAMP affected the expression and distribution of TLR in tissue mast cells [19]. CAMP can be used as a powerful inducer of CCL3 and ROS generation [19]. Research by Li et al. [20] showed that CAMP could promote epithelial and smooth muscle-like differentiation of adipose stem cells through Wnt/β-Catenin and nuclear factor-kappaB (NF-κB) pathways.

In this study, we analyzed CAMP expression and methylation levels in OA chondrocytes through the GSE117999 dataset and GSE48422 dataset, and analyzed the effects of CAMP methylation on chondrocyte oxidative stress and inflammation level in vitro, as well as the impact on chondrocyte proliferation and apoptosis. It is of great significance for further elucidating the mechanism of OA, and it also provides a basis for early diagnosis and treatment of OA.

## Materials and methods

### Datasets

Both the GSE117999 dataset and GSE48422 dataset [21] can be obtained from the Gene Expression Omnibus (https://www.ncbi.nlm.nih.gov/geo/) database. The GSE117999 dataset contains transcript data from OA and non-OA cartilage tissue. The GSE48422 dataset contains gene methylation levels in OA patients and non-OA cartilage tissues.

### Cell culture

HC-OA cells were purchased from Cell Applications (San Diego, CA, USA), and Human Chondrocytes-articular (HC-A) cells were purchased from ScienCell (San Diego, CA, USA). HC-OA cells and HC-A cells were cultured, supplemented with 10% fetal bovine serum (FBS) (Sigma-Aldrich, St. Louis, MO, USA), 1% penicillin-streptomycin (ThermoFisher Scientific, Waltham, MA) Chondrocyte growth medium (PromoCell, Heidelberg, Germany) at 37 °C in a humidified incubator containing 5% CO_2_.

### Cell transfection and AZA treatment

In order to study the effect of CAMP on the proliferation and apoptosis of chondrocytes, we used small hairpin RNA to silence *CAMP* gene expression (sh-CAMP) and no template control (sh-NC) and no transfection group (Control) as controls. According to the manufacturer’s instructions, HC-OA cells (5 × 105 cells/well) were transfected with Lipofectamine 3000 (Life Technologies, Gaithersburg, MD, USA) and incubated in a medium containing 10% FBS for 48 h. The detailed process of AZA processing has been previously reported [22]. Quantitative reverse transcription-PCR (qRT-PCR) and western blot were used to detect transfection efficiency.

### Cell Counting Kit-8 assay

CCK-8 assay was used to detect HC-OA cell proliferation ability. HC-OA cells were seeded in 96-well plates (1.0 × 10^4^ cells/well), incubated at 37 °C for 0, 24, 48, and 72 h, and then added to CCK-8 (Beyotime, Haimen, China) and incubated for 2 h. A microplate reader (BIOTEK, VT, USA) was used to detect the optical density (OD) value at 450 nm.

### ROS level detection

The cultured HC-OA cells were washed with 1 × phosphate-buffered solution (PBS), and the Reactive Oxygen Species Assay Kit (Sigma-Aldrich, Minneapolis, USA) was used to detect ROS levels according to the supplier’s instructions. Three replicate wells in each group, a microplate reader (BIOTEK, VT, USA) was used to detect the absorbance at 485/535 nm.

### qRT-PCR

The total RNA in HC-OA cell was extracted by TRIzol reagent (Invitrogen, Carlsbad, CA). The concentration of the extracted RNA was determined by NanoDrop 2000 (Thermo Fisher Scientific). Reverse transcription kit (Takara, Dalian, China) was used to reverse transcribe the extracted total RNA into cDNA. QRT-PCR was performed on an Applied Biosystems StepOnePlus Real-time PCR system (Applied Biosystems, Foster City, CA, USA) using SYBR Green Real-time PCR Master Mix (Toyobo, Osaka, Japan) according to the manufacturer’s protocol. Glyceraldehyde-3-phosphate dehydrogenase (GAPDH) was served as endogenous control. The *CAMP* mRNA primer sequences are 5’-GAA GAC CCA AAG GAA TGG CC-3’ (forward) and 5’-TCA GAG CCC AGA AGC CTG AG-3’ (reverse). GAPDH primer sequences: 5’-GAA GGT GAA GGT CGG AGT C-3’ (forward), 5’-GAA GAT GGT GAT GGG ATT TC-3’ (reverse). The relative expression of *CAMP* mRNA was calculated using the 2^−∆∆Ct^ method.

### Western blot

HC-OA cells were lysed with radioimmunoprecipitation assay (RIPA) buffer (Beyotime, Shanghai, China) for half an hour and centrifuged at 17,000 × g for 45 min at 4 °C. Bicinchoninic acid (BCA) protein assay kit (Beyotime, Shanghai, China) was used to detect protein concentration. Then sodium dodecyl sulfate-polyacrylamide gel electrophoresis (SDS-PAGE) was used to separate the proteins, and proteins were transferred to polyvinylidene fluoride (PVDF) membrane, and then used Tween–Tris-buffered saline (TTBS) containing 5% skim milk to seal the PVDF membrane at room temperature. Then, anti-CAMP (#ab80895, 1:1000, Abcam, Cambridge, UK) and anti-GAPDH (#ab181602, 1:1000, Abcam, Cambridge, UK) were incubated with PVDF membrane overnight at 4 °C. The membrane was then washed 3 times with TTBS and incubated with a secondary antibody conjugated with horseradish peroxidase at room temperature for 2 h. The blot was visualized with an enhanced chemiluminescence (ECL) kit (Santa Cruz Biotechnology) and scanned by ChemImager 5500 V2.03 software. The relative integrated density value (IDV) was calculated using GAPDH as an internal control.

### CpG methyltransferase treatment and luciferase reporter assay

The pCpGfree-CAMP (− 828/ − 1) plasma was treated by M.SssI or M.HpaII methyltransferase, and then the methylation was confirmed by restriction enzyme HpaII. The HC-OA cells were seeded in 6-well plates. When the cells were cultured to 60–90% confluence, methylated or unmethylated plasmids were transfected into HC-OA cells by Lipofectamine 3000 (Invitrogen, California, USA) according to the manufacturer’s recommendations. Forty-eight hours after transfection, the medium was collected, and 20 μl aliquots were added into the 96-well plate. A 100 μl Quanti-Luc™ (InvivoGen, San Diego, CA, USA) was injected into each well and read the luciferase activity.

### Statistical analysis

GraphPad Prism 8.0 (GraphPad Software, San Diego, CA) was used for statistical analysis of the data in this study. Two-tailed Student’s *t* test and analysis of variance (ANOVA) were used to the difference between groups. *p* < 0.05 was considered to have statistical significance.

## Results

### The expression of CAMP in cartilage tissue of OA patients was upregulated, and the level of methylation was downregulated

**Fig. 1:**
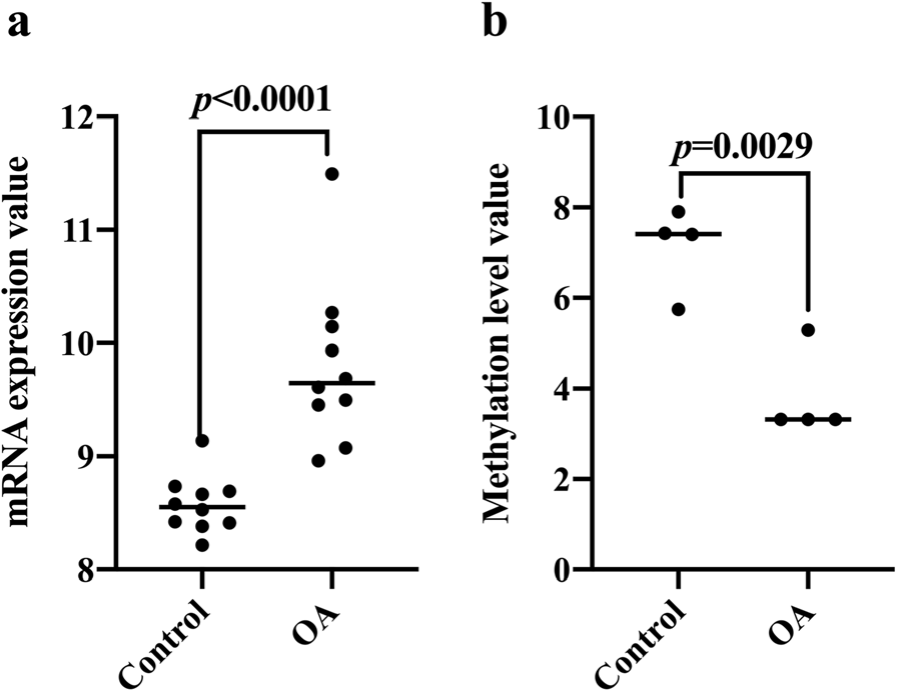
Transcriptome and methylation level detection in OA patients. **a** Analysis of the relative expression level of *CAMP* mRNA in the GSE117999 dataset (*n*_control_ = 10, *n*_OA_ = 10). **b** The methylation level of *CAMP* gene in the GSE48422 dataset (*n*_control_ = 4, *n*_OA_ = 4)

The analysis of the GSE117999 dataset shows that CAMP was upregulated in the articular cartilage of patients with OA (Table 1, Fig. 1a). The analysis of the GSE48422 dataset [21] showed that the level of CAMP methylation was downregulated (Table 2, Fig. 1b).

**Table 1:** Top 15 upregulated and downregulated gene in arthritic samples from patients with and without osteoarthritis ranked by logFC after GEO2R analysis

GENE_SYMBOL	Adj. *p* value	logFC	Up/Down
CRB1	1.52E–04	1.01580843	Up
ZSCAN12	1.89E–03	1.0271285	Up
LEO1	1.21E–04	1.03342688	Up
ESR1	4.03E–04	1.04997982	Up
FAM20A	2.36E–03	1.05411929	Up
ZNF354B	2.06E–04	1.05630409	Up
CCR10	1.15E–03	1.06099425	Up
INCENP	1.21E–04	1.18121011	Up
CAMP	3.24E–03	1.23685287	Up
THAP5	1.31E–05	1.27811954	Up
SMNDC1	8.17E–04	1.29092563	Up
HOXC8	9.69E–05	1.30931826	Up
LCE2B	3.73E–04	1.34166775	Up
FAM90A1	2.87E–03	1.40391379	Up
QRFPR	4.59E–04	1.57552192	Up
SLC38A6	1.41E–03	− 1.64465134	Down
PCDHB10	6.24E–04	− 1.43430506	Down
HLA-DRB4	1.21E–04	− 1.4061153	Down
TOLLIP	8.09E–04	− 1.31133046	Down
HLA-DQA2	9.20E–04	− 1.29212829	Down
TP53TG3D	1.99E–03	− 1.25442713	Down
CHST1	2.18E–03	− 1.1748763	Down
ANO8	2.34E–03	− 1.16784996	Down
TRIM78P	2.18E–03	− 1.10578964	Down
ADHFE1	1.66E–03	− 1.07972333	Down
DUT	3.48E–03	− 1.07647613	Down
SS18L1	2.18E–03	− 1.07511907	Down
FRMD8	1.72E–04	− 1.06645789	Down
FAM184B	2.18E–03	− 1.06047291	Down
TMEM259	9.41E–04	− 1.05326043	Down

*FC*, fold change

**Table 2:** Top 20 hypomethylated genes in arthritic and non-arthritic knee cartilage samples ranked by logFC after GEO2R analysis

GENE_SYMBOL	*p* value	logFC	Up/Down
GUCY2D	0.00902988	4.21533489	Up
GPR23	0.01044718	4.10888772	Up
LMBR1L	0.0050466	4.06301737	Up
MOBP	0.02433125	3.81893625	Up
RASSF8	0.00004736	3.71186833	Up
SLC6A5	0.02136651	3.49741335	Up
ING4	0.00371036	3.40976791	Up
CDK5	0.01690306	3.39845271	Up
hsa-mir-29b-2	0.00810529	3.39799728	Up
PITX1	0.03355549	3.31112165	Up
TLE6	0.02379696	3.31057234	Up
IL22	0.02971686	3.29164562	Up
CDIPT	0.02299476	3.24065781	Up
FLJ10986	0.0034974	3.21953344	Up
KIAA1970	0.02280297	3.1642642	Up
GDF7	0.01267419	3.14401884	Up
CAB39L	0.00421281	3.13792295	Up
CHAF1B	0.00197712	3.13636909	Up
C15orf2	0.01644456	3.07462778	Up
TMPRSS6	0.04829902	3.06043658	Up
NDUFV3	0.01618486	− 3.45593987	Down
CINP	0.01970577	− 3.40575442	Down
ACOX3	0.0123907	− 3.09168387	Down
KLK15	0.00245482	− 3.01407804	Down
RP13-360B22.2	0.04529023	− 2.9067811	Down
C10orf120	0.04175249	− 2.85198326	Down
C10orf26	0.03356837	− 2.83509207	Down
ATP6AP2	0.00771311	− 2.81820993	Down
MED28	0.01218592	− 2.77282343	Down
ZDHHC16	0.0367154	− 2.75390711	Down
LOC57228	0.03643365	− 2.71534123	Down
C15orf5	0.00278977	− 2.67864013	Down
ALDH1A3	0.03431337	− 2.65248752	Down
DNMT2	0.03566104	− 2.64961214	Down
MGC41945	0.02997186	− 2.64282551	Down
ASF1B	0.0193366	− 2.62752981	Down
CAMP	0.01873345	− 2.62260485	Down
ZNF44	0.01006365	− 2.56469726	Down
IDS	0.00377758	− 2.56033268	Down
TCN2	0.01701305	− 2.53741851	Down

*FC*, fold change

### CAMP was highly expressed in osteoarthritis articular cartilage cells

**Fig. 2:**
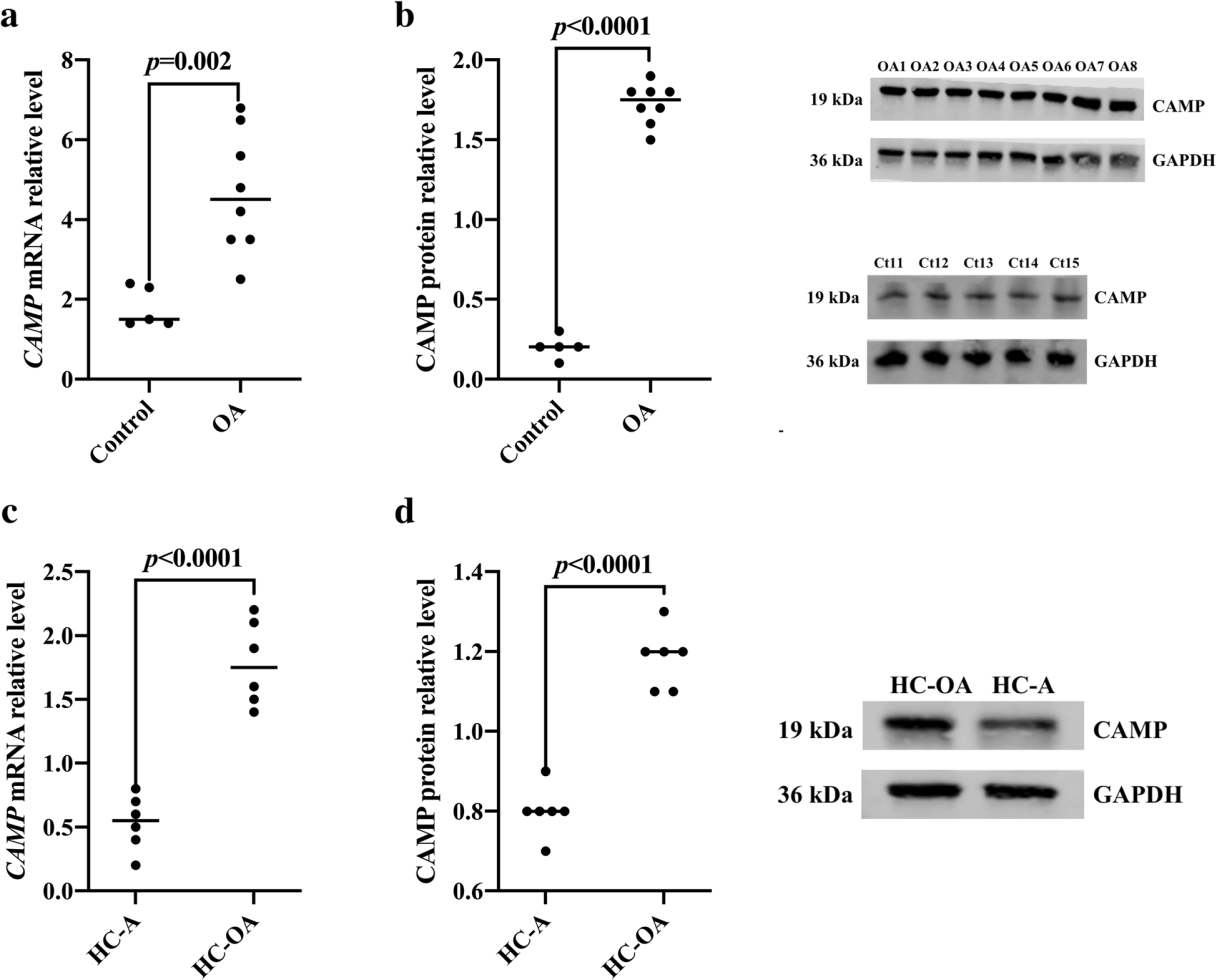
CAMP was highly expressed in osteoarthritis articular cartilage cells. **a** The expression level of *CAMP* mRNA in articular cartilage tissues of OA patients and control group were analyzed by qRT-PCR. **b** The expression level of CAMP protein in articular cartilage tissues of OA patients and control group were analyzed by western blot. **c** *CAMP* mRNA expression levels in Human Chondrocytes Osteoarthritis (HC-OA) cells and Human Chondrocytes-articular (HC-A) cells were analyzed by qRT-PCR. **d** CAMP protein expression levels in HC-OA cells and HC-A cells were detected by western blot

We analyzed the expression levels of CAMP in the cartilage tissue of 8 cases of OA patients undergoing total knee arthroplasty and 5 cases of trauma requiring high amputation (excluding OA) (Control). The results showed that the expression levels of *CAMP* mRNA and CAMP protein in the articular cartilage tissue of KOA patients were significantly higher than those of the control group (*p* = 0.002, *p* < 0.0001, Fig. 2a, b). Our analysis in Human Chondrocytes Osteoarthritis (HC-OA) cells and Human Chondrocytes-articular (HC-A) cells showed that the *CAMP* mRNA level and CAMP protein level in HC-OA cells were significantly higher than those in HC-A cells (*p* < 0.001, *p* < 0.0001, Fig. 2c, d).

### Silencing *CAMP* gene inhibited the proliferation of osteoarthritis articular cartilage cells and promoted their apoptosis

**Fig. 3:**
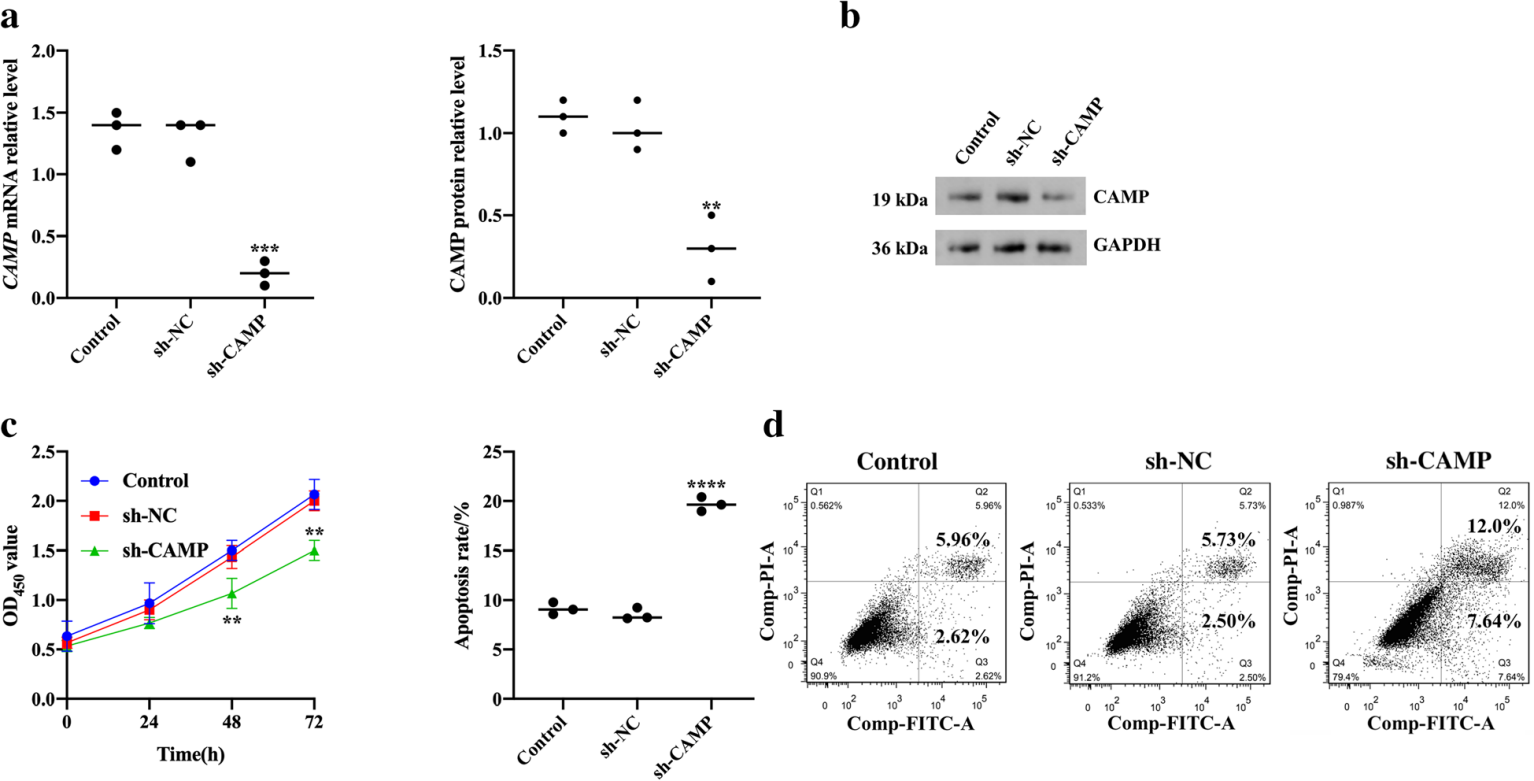
Silencing *CAMP* gene inhibited the proliferation of osteoarthritis articular cartilage cells and promoted their apoptosis. **a** The expression level of *CAMP* mRNA in Control, sh-NC, and sh-CAMP-transfected HC-OA cells was detected by qRT-PCR. **b** the expression level of CAMP protein in Control, sh-NC, and sh-CAMP-transfected HC-OA cell was detected by western blot. **c** The cell proliferation ability of Control, sh-NC, and sh-CAMP-transfected HC-OA cells was detected by CCK-8. **d** The apoptotic ability of Control, sh-NC, and sh-CAMP-transfected HC-OA cells was detected by flow cytometry. ^**^p < 0.01, ^***^p < 0.001, ^****^p < 0.0001, compare with Control

In order to study the role of *CAMP* gene in the proliferation and apoptosis of chondrocytes in OA patients, we used small hairpin RNA to silence *CAMP* gene (sh-CAMP) and no template control (sh-NC) and no transfection group (Control) as control. QRT-PCR and western blot results confirmed that *CAMP* gene was successfully knocked out in HC-OA cells (*p* < 0.001, *p* < 0.01; Fig. 3a, b). CCK-8 assay results showed that compared with the Control, the proliferation ability of HC-OA cells was significantly inhibited after sh-CAMP transfection (*p* < 0.01, Fig. 3c). Flow cytometry detection of cell apoptosis showed that the apoptotic rate of HC-OA cells after sh-CAMP transfection was significantly higher than that of the Control (*p* < 0.01, Fig. 3d).

### *CAMP* gene methylation inhibited ROS levels and inflammatory response levels in osteoarthritis articular cartilage cells

**Fig. 4:**
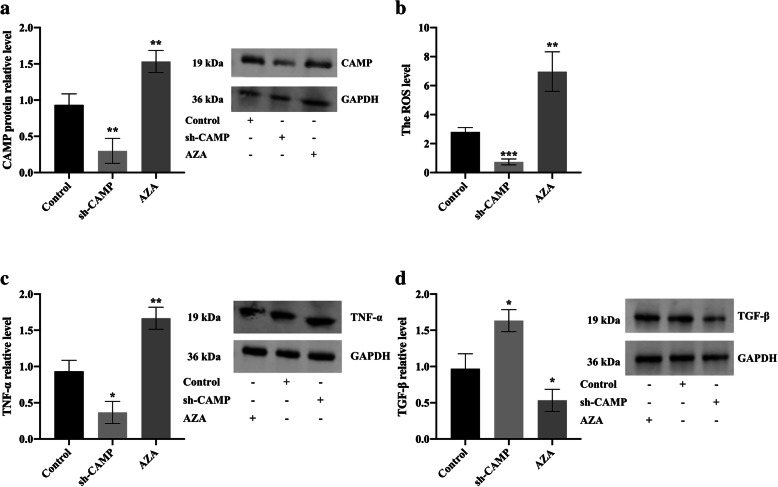
*CAMP* gene methylation inhibited ROS levels and inflammatory response levels in osteoarthritis articular cartilage cells. **a** The CAMP protein expression level in Control, sh-CAMP-transfected HC-OA cells, and 5-μM AZA-treated HC-OA cells were analyzed by western blot. **b** The comparison of the ROS levels in Control, sh-CAMP-transfected HC-OA cells, and 5-μM AZA-treated HC-OA cells. **c** The TNF-α protein expression levels in Control, sh-CAMP-transfected HC-OA cells, and 5-μM AZA-treated HC-OA cells were analyzed by western blot. **d** The TGF-β protein expression levels in Control, sh-CAMP-transfected HC-OA cells, and 5-μM AZA-treated HC-OA cells were analyzed by western blot. ^*^p < 0.05, ^**^p < 0.01, ^***^p < 0.001, compared with Control

In order to further study the effect of CAMP methylation on the level of oxidative stress and inflammatory response levels in HC-OA cells, we set up 3 groups, namely Control, sh-CAMP-transfected HC-OA cell group (sh-CAMP), and 5-μM AZA treatment group. First, western blot results showed that compared with the Control, the CAMP protein expression in HC-OA cells was significantly downregulated after sh-CAMP transfection, and CAMP expression in HC-OA cells was significantly increased after AZA treatment (Fig. 4a). After transfection with sh-CAMP, the level of ROS in HC-OA cells decreased significantly, while the level of ROS in HC-OA cells increased after AZA treatment (Fig. 4b). Western blot results showed that compared with Control, TNF-α levels in HC-OA cells were significantly decreased after sh-CAMP transfection, and TNF-α levels were significantly increased after AZA treatment (Fig. 4c). The level of TGF-β in HC-OA cells was significantly increased after sh-CAMP transfection, and AZA treatment downregulated the expression of TGF-β (Fig. 4d). These results showed that *CAMP* gene methylation downregulated the expression of CAMP in HC-OA cells and inhibited the level of oxidative stress and inflammatory response levels.

### *CAMP* gene methylation inhibited the proliferation of osteoarthritis articular cartilage cells and promoted their apoptosis

**Fig. 5:**
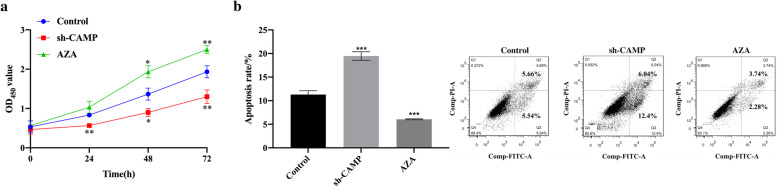
*CAMP* gene methylation inhibited the proliferation of osteoarthritis articular cartilage cells and promoted their apoptosis. **a** The cell proliferation ability of Control, sh-CAMP-transfected HC-OA cells, and 5-μM AZA-treated HC-OA cells were detected by CCK-8. **b** The apoptotic rate of Control, sh-CAMP-transfected HC-OA cells and 5-μM AZA-treated HC-OA cells were detected by flow cytometry. ^*^p < 0.05, ^**^p < 0.01, ^***^p < 0.001, compared with Control

In order to further study the effect of *CAMP* gene methylation on the proliferation and apoptosis of HC-OA cells, we set up 3 groups, namely Control, sh-CAMP, and AZA. Western blot analysis showed that, compared with Control, the CAMP protein in the sh-CAMP transfection group was significantly downregulated, and the CAMP protein expression level was significantly increased after AZA treatment (Fig. 4a). CCK-8 assay results showed that compared with Control, the proliferation ability of HC-OA cells in the sh-CAMP transfection group was inhibited, and the proliferation ability of HC-OA cells was significantly increased after AZA treatment (Fig. 5a). The results of flow cytometry showed that compared with Control, the apoptotic rate of HC-OA cells in the sh-CAMP transfection group was significantly increased, and the apoptotic rate of HC-OA cells was significantly decreased after AZA treatment (Fig. 5b). These results showed that *CAMP* gene methylation inhibited the proliferation of osteoarthritis articular cartilage cells and promoted their apoptosis.

### DNA methylation directly downregulated the activity of *CAMP* gene promoter

**Fig. 6:**
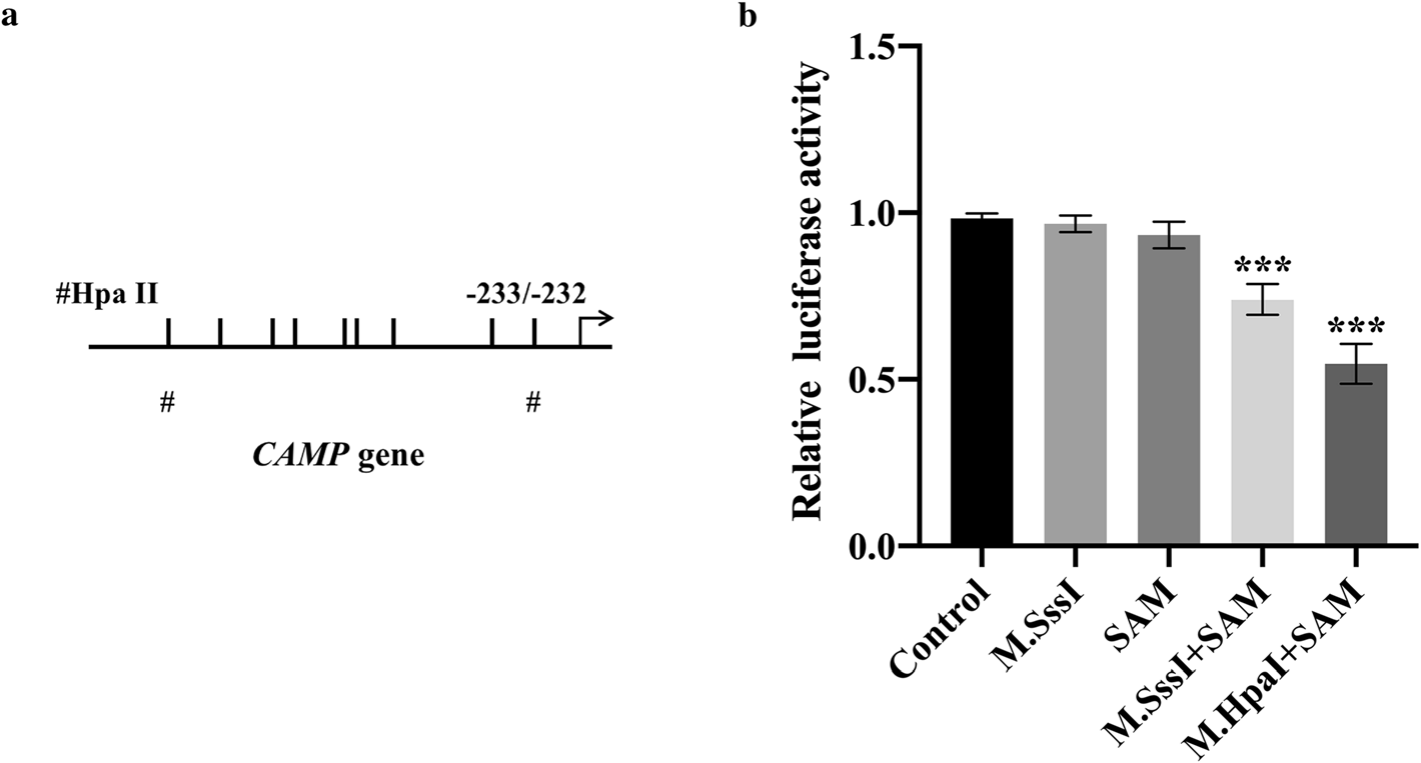
Direct downregulation of *CAMP* gene promoter activity by DNA methylation. **a** A schematic illustration of the *CAMP* gene promoter. The major transcriptional start site is indicated by the arrow at position +1. Upper tick marks indicate individual CpG sites in CAMP gene promoter. The symbols of “#” represent HpaII sites. **b** Luciferase activities of unmethylated and methylated *CAMP* gene promoter constructs in HC-OA cells. *SAM*, S-adenosylmethionine. ^***^p < 0.001, compared with Control

In order to further verify whether DNA methylation can downregulate the activity of the *CAMP* gene promoter, a pCpGfree-basic-Lucia construct without CpG dinucleotides was used for luciferase reporter assay. S-adenosylmethionine (SAM) is used as the methyl donor for the enzymatic reaction, and pCpGfree-CAMP (− 828/ − 1) was treated with M.SssI and M.HpaII methyltransferase. Untreated, M.SssI-treated or SAM-treated plasmids were used as controls. Then the methylated or unmethylated plasmid was transfected into the HC-OA cells, and the luciferase activity was measured. The results showed that higher promoter activity was detected in HC-OA cells transfected with the unmethylated pCpGfree-CAMP (− 828/ − 1) construct. Compared with the unmethylated construct, when the pCpGfree-CAMP (− 828/ − 1) construct treated with M.SssI was transfected into HC-OA cells, the promoter activity was significantly reduced (Fig. 6). In addition, HC-OA cells transfected with the pCpGfree-CAMP (− 828/ − 1) construct were only methylated by M.HpaII methyltransferase treatment and also showed a similar downregulation of promoter activity (Fig. 6). This indicates that the CpG site of M.HpaII in the CAMP promoter is important for promoter activity regulation, and the scattered methylation on the CpG site may not be able to effectively reduce the activity of the gene promoter. We can also know that the CpG site (− 233/ − 232) closest to the main transcription start site of *CAMP* is important for transcription regulation. These results were consistent with the results of AZA treatment inducing CAMP expression (Figs. 4 and 5). In summary, these results indicated that DNA methylation directly downregulated the activity of the *CAMP* promoter in HC-OA cells.

## Discussion

In this study, we extracted and analyzed the differentially expressed genes and methylation levels in chondrocytes from public databases, and found that CAMP (also known as LL-37) was upregulated in the articular cartilage of patients with OA, the methylation level of *CAMP* gene was downregulated. We found that CAMP protein expression was upregulated in articular cartilage tissue of OA patients and HC-OA cells. After silencing the expression of the *CAMP* gene, we found that the proliferation of HC-OA cells was inhibited, accompanied by a significant increase in the apoptotic rate. After demethylation, the level of ROS and inflammation in HC-OA cells increased significantly, the cell proliferation ability was significantly enhanced, and the apoptosis rate was significantly reduced. Therefore, we speculated that *CAMP* gene promoter methylation induced chondrocyte apoptosis by inhibiting ROS levels and inflammatory response levels.

Current research evidence shows that risk factors for OA include genetics, inflammation, environmental factors, age, stress stimulation, etc. [23, 24]. In recent years, more and more studies have found that epigenetic modification plays an important role in the pathogenesis of OA. Epigenetic modification works by regulating gene transcription or post-transcriptional regulation, including DNA methylation, histone modification, chromosome remodeling, and non-coding RNAs (ncRNAs) [6, 25, 26]. Usually, DNA methylation occurs in cytosine, guanine, and nucleotides (CpG), which is mainly manifested as the phenomenon of conversion of cytosine to 5-methylcytosine [27, 28]. The research on DNA methylation function mainly focuses on the transcription start site of genes [29]. Methylation of the promoter region can inhibit gene expression; on the one hand, methylation of the CpG site directly interferes with the binding of transcription factors to DNA in the regulatory region; on the other hand, methylated DNA and methylated CpG binding region proteins such as MeCP2 specifically bind to form a complex, which restricts the passage of transcription factors to their binding sites, thereby inhibiting gene expression [30–32].

In this study, we found that the expression level of CAMP protein increased significantly after AZA treatment. The reason might be that the methylation level of the promoter region of *CAMP* gene decreased after AZA treatment, and the binding efficiency of *CAMP* gene transcription factors increased; as a result, the expression level of CAMP increased significantly. At present, researchers have tried to use AZA for in vitro intervention of OA chondrocytes. For example, Alvarez-Garcia et al. [33] treated TC28 cells with AZA and the expression of atonal bHLH transcription factor 8 (ATOH8) and T-box transcription factor 4 gene (TBX4) increased significantly. In addition, some researchers found that the DNA methylation level of the inducible nitric oxide synthase (iNOS) enhancer-5.8 kb CpG site decreased after AZA treatment, and the iNOS expression level increased; the methylation of the iNOS enhancer could inhibit the cell cycle process by downregulating NF-κB level and reduce the pro-inflammatory response, which had important therapeutic significance for OA [34].

In this study, we found that CAMP was highly expressed in articular cartilage cells of OA patients. Silencing the expression of *CAMP* gene inhibited the proliferation of osteoarthritis articular cartilage cells and promoted their apoptosis, suggesting that CAMP may play an important role in the pathogenesis of osteoarthritis. Yu et al. [35] showed that CAMP inhibited inflammation and promoted bone formation of bone marrow stromal cells (BMSC) through purinergic receptor P2X 7 (P2RX7) and mitogen-activated protein kinase (MAPK) signaling pathway. Further, in vitro studies had found that *CAMP* gene methylation inhibited the level of ROS and TNF-α in chondrocytes, promoted the level of TGF-β expression, inhibited the proliferation of chondrocytes, and promoted their apoptosis. The results of Kuensaen et al. [36] found that high levels of CAMP promote the expression of downstream pro-inflammatory cytokines (especially IL17A), which was related to the pathogenesis of inflammatory arthritis. Hu et al. [37] found that CAMP regulated the production of inflammatory cytokines such as TNF-α and inhibited cell apoptosis. The results of this study suggested that the expression of *CAMP* gene and methylation in promoter region might be involved in the occurrence of osteoarthritis from the level of oxidative stress and the expression of inflammatory factors.

There are some limitations in this study. First of all, the treatment of the articular cartilage tissue samples used for analysis is unclear, and whether there are drugs that affect the expression of inflammatory factors in tissue is unclear. Secondly, the expression and methylation of *CAMP* gene may lead to the occurrence of OA through other signaling pathways, which was not confirmed in this study. In addition, the results of this study need to be further confirmed in vivo.

## Conclusion

In this study, we found that *CAMP* gene promoter methylation induces chondrocyte apoptosis by inhibiting ROS levels and inflammatory response levels, which is a potential target for OA therapy.

## Data Availability

The datasets during and/or analyzed during the current study available from the corresponding author on reasonable request.
